# Successful treatment of lipofibromatosis-like neural tumor of the lumbar spine with an NTRK-fusion inhibitor

**DOI:** 10.1186/s13569-020-00136-6

**Published:** 2020-08-06

**Authors:** Megan Dupuis, Yulei Shen, Christian Curcio, Jeanne M. Meis, Wei-Lien Wang, Behrang Amini, Laurence Rhines, Jacquelyn Reuther, Angshumoy Roy, Kevin E. Fisher, Anthony P. Conley, J. Andrew Livingston

**Affiliations:** 1 Division of Cancer Medicine, University of Texas MD Anderson Cancer Center, Houston, TX USA; 2 Department of Pathology & Immunology, Baylor College of Medicine and Texas Children’s Hospital, Houston, TX USA; 3 Department of Pathology, University of Texas MD Anderson Cancer Center, Houston, TX USA; 4 Department of Diagnostic Imaging, University of Texas MD Anderson Cancer Center, Houston, TX USA; 5 Department of Neurosurgery, University of Texas MD Anderson Cancer Center, Houston, TX USA; 6 Department of Sarcoma Medical Oncology, University of Texas MD Anderson Cancer Center, 1515 Holcombe Blvd. Unit 0450, Houston, TX 77030 USA; 7 Department of Pediatrics, University of Texas MD Anderson Cancer Center, Houston, TX USA

**Keywords:** Lipofibromatosis-like neural tumors, LPF-NT, NTRK, Entrectinib

## Abstract

**Background:**

Lipofibromatosis-like neural tumors (LPF-NT) are a newly identified class of rare mesenchymal neoplasms. Current standard of care therapy is surgical resection alone; there are no chemotherapies or molecular targeted therapies that have been shown to be effective in patients who are not surgical candidates due to either tumor bulk or location. Most LPF-NT harbor NTRK fusions, although the therapeutic significance of these fusions has not been previously demonstrated in this malignancy. Here, we present the first case of a patient with surgically-unresectable LPF-NT successfully treated with medical therapy, specifically the TRK fusion-protein inhibitor entrectinib.

**Case presentation:**

The patient is a 21 year old man with no co-morbidities who presented for evaluation due to intermittent abdominal pain and was found to have a mass spanning from T12-L2. Biopsy revealed a mesenchymal spindle cell neoplasm and S100 positivity pointed to possible nerve sheath origin. The sample was ultimately found to have an LMNA-NTRK1 fusion, confirming the diagnosis of LP-NFT. Unfortunately, due to the bulk and location of the tumor, surgery was felt to be exceptionally morbid and the patient was treated in a clinical trial with the NTRK inhibitor entrectinib. Surprisingly, he had such a robust clinical response that he was ultimately deemed a surgical candidate and he was successfully taken to surgery. Post-operative pathology revealed > 95% necrosis, demonstrating exceptional sensitivity to the targeted therapy. The patient remains NED and on entrectinib 12 months post-operatively.

**Conclusions:**

The exceptional treatment response of this patient suggests that NTRK fusions are true drivers of the disease. Thus, all patients should be evaluated for NTRK fusions using sensitive methodologies and treatment with TRK fusion-protein inhibitors should be considered in patients who are not candidates for oncologic resection.

## Background

Lipofibromatosis-like neural tumors (LPF-NT) are a rare subset of typically superficial mesenchymal neoplasms initially described in 2016 [1]. While morphologically similar to lipofibromatosis in that they contain spindle cells involving fibroadipose tissue and are positive for CD34 and SMA, they are also positive for S-100 protein which suggests neural differentiation. Importantly, LPF-NT typically contain a driver fusion protein involving TRK, a family of neurotrophic receptor tyrosine kinases [1]. This molecular alteration distinguishes this neoplasm from lipofibromatosis, which does not harbor a TRK fusion protein [1]. These tumors are locally invasive and can carry significant morbidity [1]. Currently, front-line therapy for LPF-NT is surgery alone; if resection is not an option due to tumor bulk or location, there are no validated standardized treatments. Thus, a lack of effective pre-surgical therapies represents a significant gap in the field for this newly-described tumor.

In recent years, cancer treatments have dramatically changed due to identification of new, druggable, oncogenic molecular drivers. One such target is the fusion product of the neurotrophic receptor tyrosine kinase genes *NTRK1, NTRK2,* and *NTRK3* (encoding proteins TRK1, TRK2, and TRK3, respectively) with a variety of genetic fusion partners [2]. These upstream fusion partners contain oligomerization domains (such as coiled-coil, zinc finger, or WD domains) [3, 4] or have alternate mechanisms of dimerization which activate TRK downstream signaling; more than 50 upstream partners have been identified thus far [5].

*NTRK* fusions have been identified in tumors of more than 20 histologies [5]. Certain *NTRK* fusions, like *ETV6*-*NTRK3*, are represented in > 90% of secretory breast carcinomas [6], mammary analog of secretory carcinomas (MASC) [7], congenital mesoblastic nephroma [8, 9], and infantile fibrosarcomas [10, 11]. These fusions are also found at lower incidences (5–25%) in tumors such as breast, lung, colon, and melanoma [2], leading to the 2018 basket trial in which 55 *NTRK*-fusion-positive patients with 17 different cancer types were treated with the first-generation TRK inhibitor larotrectinib [12]. This study ultimately resulted in the first tissue-agnostic US FDA approval for a molecular targeted therapy. Entrectinib, another first generation small-molecule inhibitor against TRKA/B/C, ROS1, and ALK, was recently developed. It was designed to cross the blood–brain barrier to target brain metastases [13], and demonstrated efficacy in multiple histologies including non-small cell lung cancer [14, 15] as well as activity in adults and children with solid tumors harboring *NTRK* fusions [16]. These studies have led to its accelerated approval by the US FDA for adults with *ROS*-*1* positive metastatic NSCLC and for adult and pediatric patients ≥ 12 years old with *NTRK* fusion-positive solid tumors.

Thus far, two papers have studied the incidence of *NTRK* fusion proteins in LPF-NT. In the 2016 paper which first classified this tumor [1], they describe that 10/14 patients (71%) had *NTRK*1 fusions. In the second study [17], molecular studies showed 4/5 patients with LPF-NF were positive for *NTRK1* rearrangement by FISH. Given the natural history and therapeutic implications of NTRK fusions in a subset of soft tissue sarcomas, the WHO has recently reclassified all NTRK-rearranged soft tissue sarcoma into a new provisional entity labeled NTRK-rearranged spindle cell neoplasm [18]. This new classification encompasses LPF-NT as well as other sarcomas harboring NTRK fusions. This move towards molecularly defined subtypes of soft tissue sarcoma is reflective of the field and the evolving role of subtype specific diagnoses and treatments. However, the significance and therapeutic implications of NTRK-fusions across various sarcoma subtypes may not be uniform and thus warrants specific evaluation as in our case of LPF-NT.

Taken together, the above studies suggest that most LPF-NT harbor an *NTRK* fusion protein, a viable therapeutic target. Here, we report the first case of an LPF-NT successfully treated with a TRK inhibitor prior to surgical resection, and discuss the implications for management of this rare entity.

## Case presentation

**Fig. 1:**
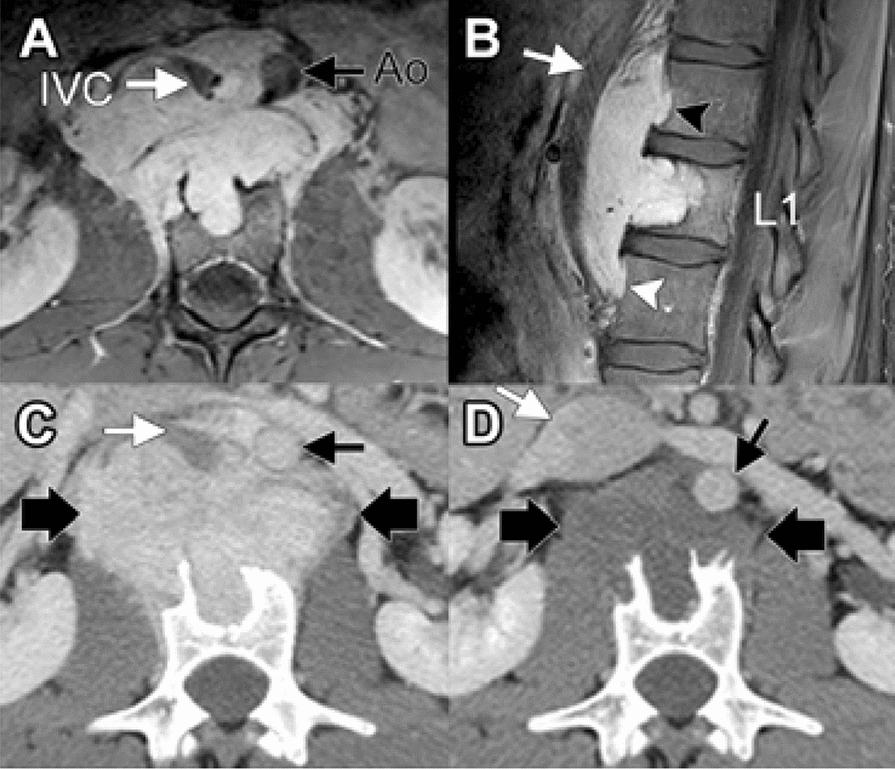
Imaging features of the lesion. **a**, **b** Axial and sagittal post-contrast MRI demonstrates an avidly enhancing lesion in the L1 pre-vertebral soft tissues. There is anterior displacement of the aorta (Ao, black arrow) and inferior vena cava (IVC, white arrows). There is invasion of the L1 vertebral body, with cephalad and caudad extension of disease and secondary pressure erosion of the ventral cortices of T12 (black arrowhead) and L2 (white arrowhead) vertebral bodies. **c** Concurrent contrast-enhanced CT image at the level of L1 shows an avidly enhancing mass (between large black arrows) with invasion of the L1 vertebral body. The CT shows rim of sclerosis in the vertebral body indicative of secondary invasion from a soft tissue mass. The aorta and inferior vena cava (black and white arrows, respectively) are anteriorly displaced. **d** Contrast-enhanced CT at end of therapy shows significant decrease in size and degree of enhancement of the mass (between large black arrows). Mass effect on the aorta and inferior vena cava (black and white arrows, respectively) has also decreased

The patient is a 21-year-old young man without significant past medical history. He initially presented with a complaint of intermittent abdominal pain, and a CT scan revealed an abnormal paraspinal lesion. He underwent MRI which demonstrated the lesion extending from T12-L2 and measured 8.7 cm × 3.9 cm × 6.9 cm (Fig. 1a, b).

**Fig. 2:**
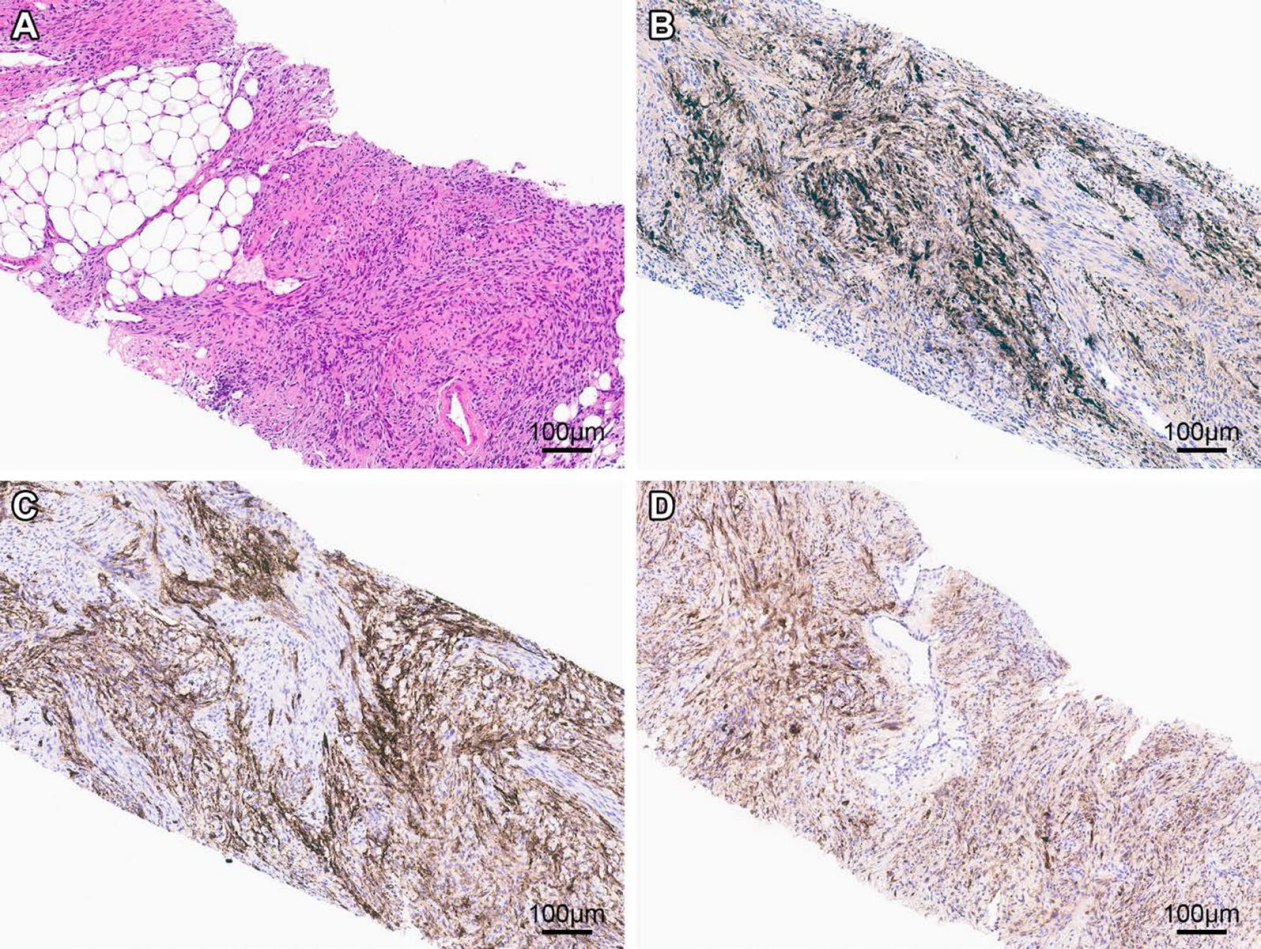
Pre-treatment biopsy. **a** H&E sections demonstrate monotonous, bland, spindle cell proliferation admixed with mature fat and occasional ectatic blood vessel. Immunohistochemical studies reveal that areas of the tumor cells had reactivity for **c** S-100, **c** CD34, and **d** pan-trk. Measurement bars = 100 µm

Subsequent biopsy revealed a spindle cell mesenchymal tumor with monotonous, bland spindle cells admixed with mature fat and occasional ectatic blood vessels (Fig. 2). Neither necrosis nor brisk mitotic activity were noted. IHC studies demonstrated positivity for CD34, pan-TRK, S-100 and SMA (not shown); the tumor was negative for Pankeratin cocktail, STAT6, DOG1, desmin, TLE1, panmelanocytic cocktail, and Sox10. Scattered cells labeled for SATB2 and H3K27me3. The tumor was negative for *MDM2* amplification or *FUS* gene rearrangement using fluorescent in situ hybridization (FISH).

**Fig. 3:**
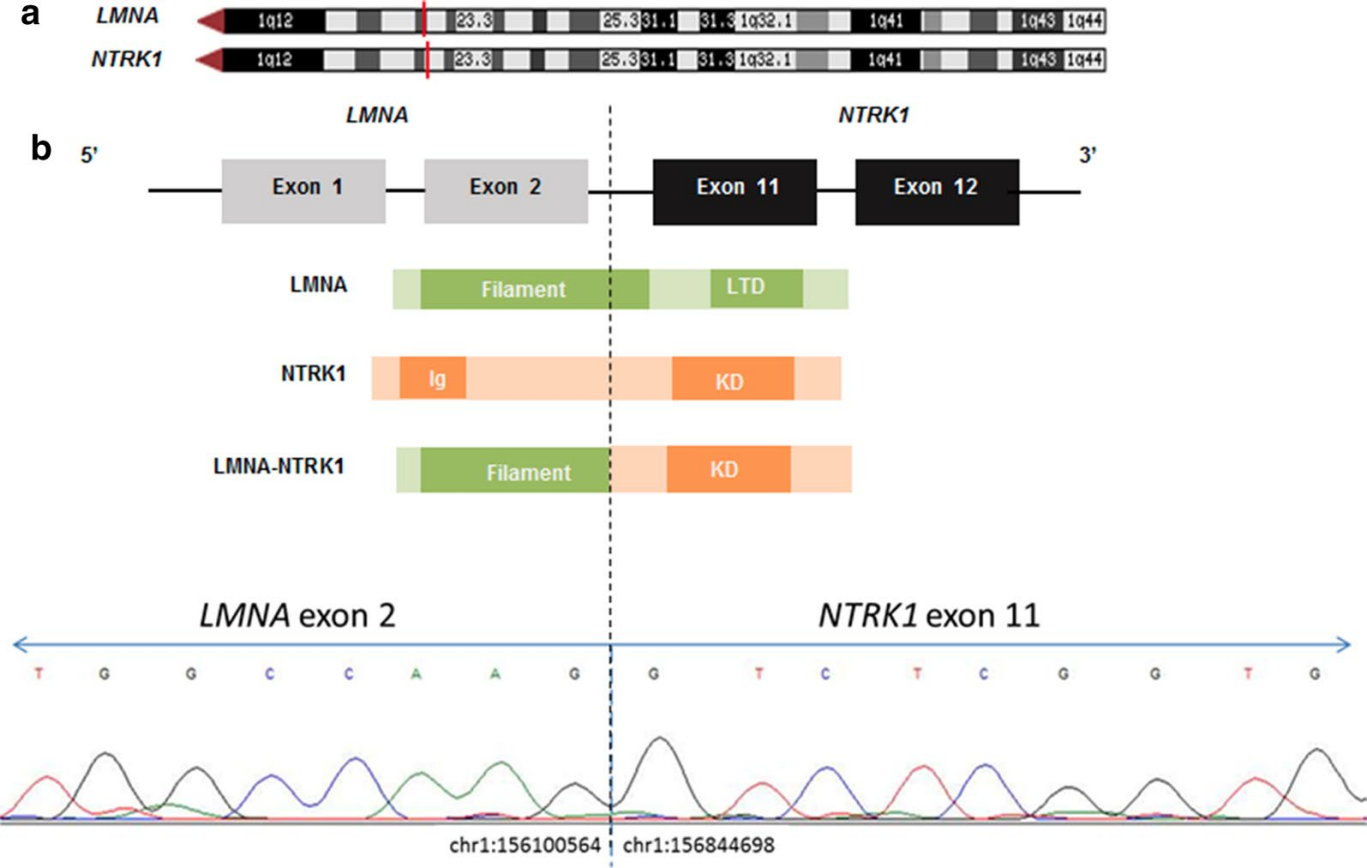
LMNA-NTRK1 fusion confirmed by RT-PCR. **a** Schematic diagram showing the predicted in-frame LMNA-NTRK1 fusion protein joining the 5′ LMNA filament domain to an intact 3′ NTRK1 tyrosine kinase domain (KD). The red dashed lines denote the 1q chromosomal positions of *LMNA* and *NTRK1*, respectively. **b** RT-PCR Sanger sequencing trace confirming the fusion breakpoint at chr1:156100564 (*LMNA,* NM_005572.3, exon 2) and chr1:156844698 (*NTRK1*, NM_002529.3, exon 11). LTD, lamin tail domain; Ig, Tyrosine-protein kinase receptor C2 Ig-like domain

The S-100 staining raised the possibility of a peripheral nerve sheath origin. Given the pan-TRK immunoreactivity, the diagnosis of LPF-NT was considered; however, the site was unusual as it was not superficial. Thus, further molecular testing was performed. A custom-designed, clinically-validated anchored multiplex PCR-based targeted next-generation sequencing (NGS) RNA fusion panel that covers 485 exons from 81 genes and is optimized for formalin-fixed, paraffin-embedded (FFPE) samples, revealed a fusion between exon 2 of *LMNA* (NM_005572.3; chr1:156100564) and exon 11 of *NTRK1* (NM_002529.3; chr1:156844698), predicted to encode an in-frame LMNA-NTRK1 fusion protein retaining the C-terminal kinase domain of NTRK1 (Fig. 3a). The fusion transcripts were confirmed with orthogonal RT-PCR and Sanger sequencing (Fig. 3b). Identification of this fusion reclassified the putative diagnosis to an S-100 positive LPF-NT of the spine.

**Fig. 4:**
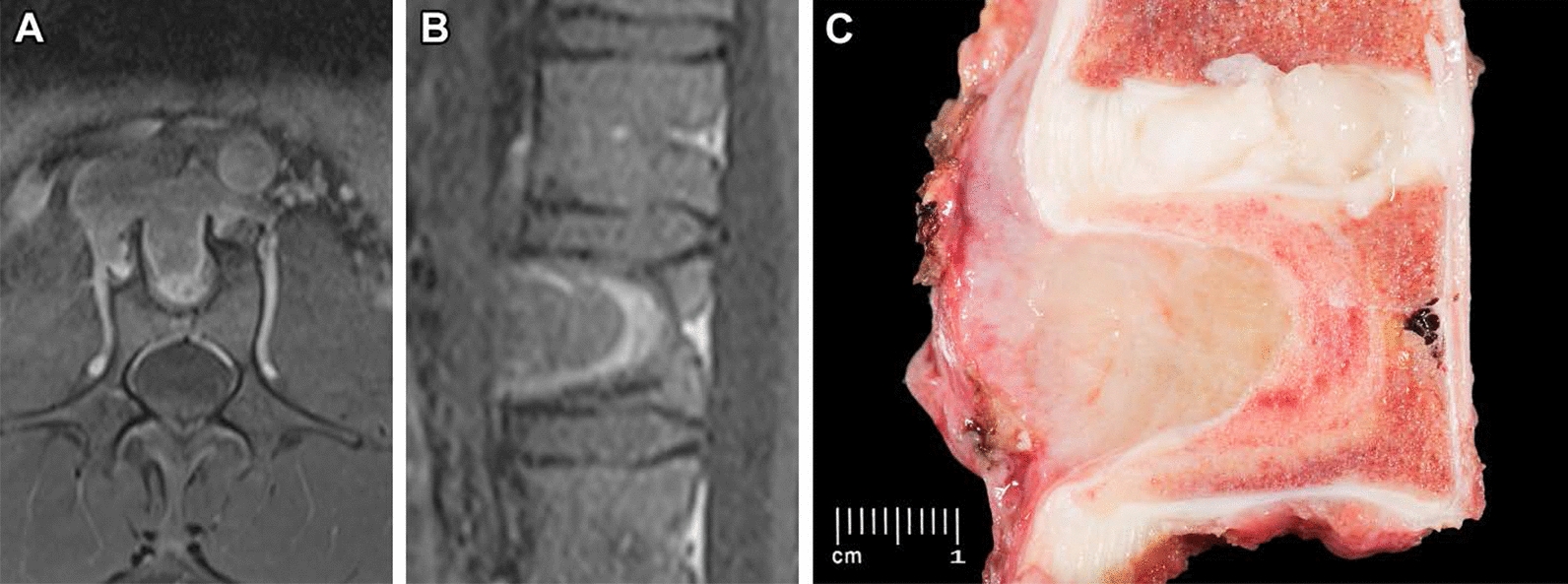
Post treatment. Contrast-enhanced MR images demonstrating mass involving vertebrae (**a**) axial and (**b**) sagittal showing decrease in the size of the lesion compared to baseline (Fig. 1). **c** Gross specimen (sagittal) demonstrating a tan-white pink mass involving the paraspinal soft tissue and protruding into the underlying vertebrae body associated with sclerosis. Measurement bar = 1 cm

**Fig. 5:**
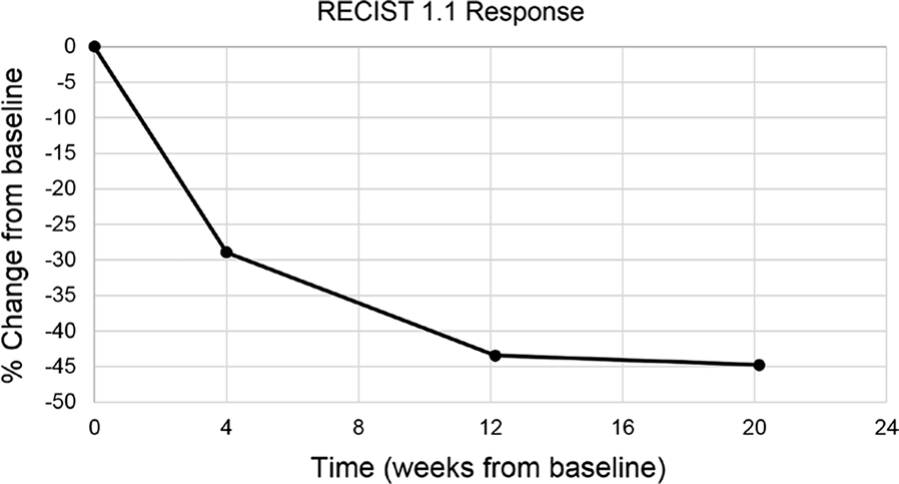
Response to therapy as assessed by RECIST 1.1. Response plateau was achieved at week 12, with 45% decrease in size of the lesion

Due to size and location, primary surgical resection would have been exceedingly morbid. Thus, the patient was entered into a phase II basket trial enrolling patients with *NTRK* 1/2/3, ROS1, or ALK gene rearrangements to be treated with entrectinib. He received a dose of 600 mg daily, and the only adverse effects were grade 1 diarrhea and taste alterations. Early response assessment after 1 month demonstrated a near-complete loss of enhancement and density of the mass, with the hounsfield units dropping from 200 on the initial scan to 55–60 at follow up (Figs. 1a, b, and 4). He had an excellent therapeutic response, with a 45% reduction in tumor size by RECIST criteria, measuring 4.0 × 2.8 cm (Fig. 5). Although the patient was not initially a surgical candidate due to unacceptable morbidity, his response to therapy was so exceptional that he ultimately qualified for surgical resection.

**Fig. 6:**
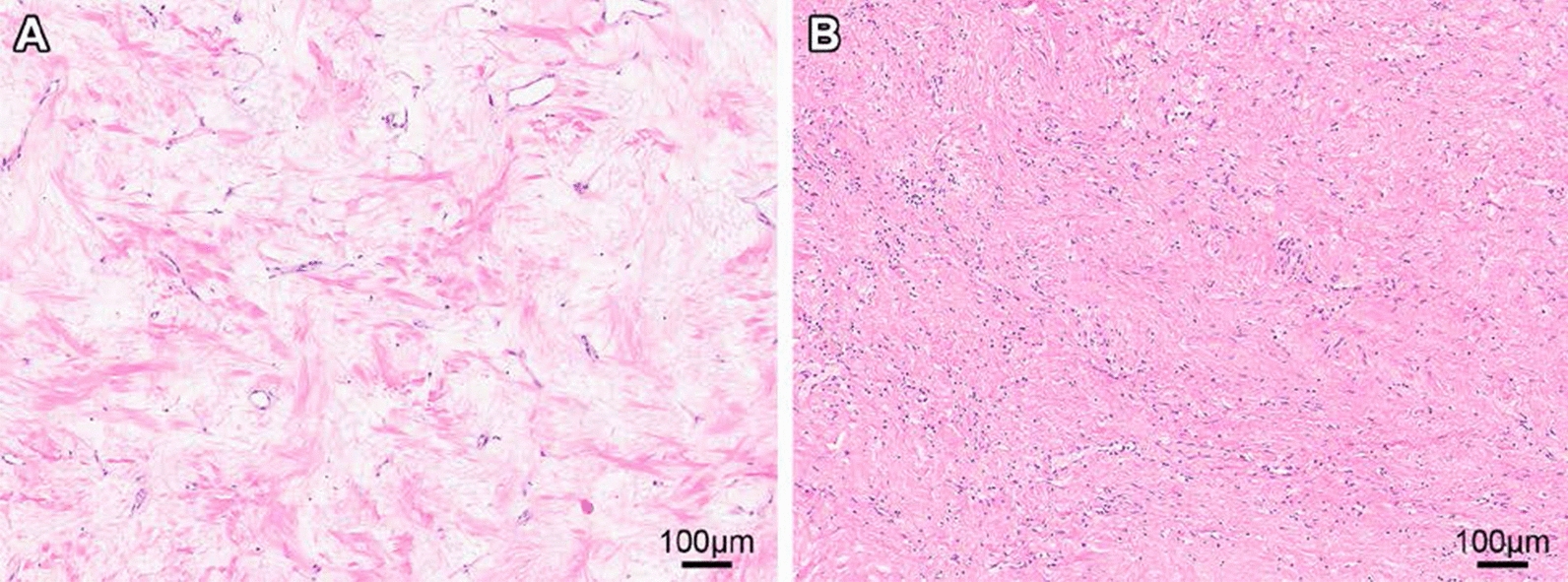
Post-treatment histological changes. **a** H&E sections demonstrate marked hyalinization and decreased cellularity with scattered foci of **b** residual tumor cells admixed with lymphocytes. Measurement bars = 100 µm

Therefore, he underwent vertebrectomy, which revealed a 7.0 × 5.2 × 3 cm mass with tan-white pink cut surface involving paraspinal soft tissue and protruding into the vertebral body, with underlying bony sclerosis. Histologically, there was extensive treatment response (> 95%) with decreased cellularity, marked hyalinization and focal areas of viable tumor cells (Fig. 6). The tumor focally involved soft tissue margins however bone margins were tumor-free. The patient has continued on entrectinib following surgery and remains without evidence of recurrence at 7 months.

## Discussion and conclusions

LPF-NT is a rare tumor of mesenchymal origin first described in 2016. Prior to then, LPF-NT was likely characterized as atypical lipofibromatosis, malignant peripheral nerve sheath tumor (MPNST), or spindle cell tumors. Given its recent classification, there is a paucity of data regarding management and outcomes. Currently, only 26 patients with LPF-NT are described in the literature [1, 17, 19]; our case, which met classification criteria for LPF-NT given positive S100 and CD34 staining, represents the 27th patient.

Importantly, his tumor was positive for a LMNA-NTRK1 fusion, which was the most common type of *NTRK* fusion seen in the initial case series [1]. The authors also separately report a single case of a patient with LPF-NT and *LMNA*-*NTRK1* fusion with lung metastasis, potentially secondary to a delay in surgical resection rather than the specific *NTRK* fusion partner. Another case report has also implicated the *LMNA*-*NTRK1* fusion in a patient with metastatic sarcoma [20]. However, the significance of specific *NTRK* fusion partners remains incompletely understood [2, 5], and their systematic classification may yield information about their biologic behavior.

In the broader sarcoma field, the significance of *NTRK* fusions has emerged. As previously mentioned, ETV6-*NTRK*3 fusions are practically pathognomonic for the diagnosis of infantile fibrosarcoma [11] and *NTRK* fusions have also been implicated as the defining feature of a subset of unclassified uterine sarcoma with spindle cell morphology [21]. However, incidence of NTRK fusions is variable between different sarcoma subtypes. Recently, position papers released from the Journal of Clinical Pathology [22] and ESMO [23] propose standardized algorithms for testing for NTRK fusions. Both propose essentially the same method: for tumors with a high incidence of NTRK fusions (such as MASC), any detection method is sufficient; however, in tumors with low incidence, an NGS panel should be used upfront with positivity confirmed via IHC. If, however, no standard NGS panel is available, then IHC screening may be used upfront, with NGS confirmation.

For poor quality specimens, a highly sensitive and versatile assay able to test for NTRK and other fusions is needed to confirm the diagnosis. Anchored multiplex PCR NGS allows for detection of multiple gene fusions in a single assay with minimal RNA input, which has high diagnostic yield in sarcomas and other spindle cell lesions [24].

As these fusions have been identified, the opportunity for targeted therapy has also become apparent. In 2015, a patient with metastatic soft-tissue sarcoma harboring an *LMNA*-*NTRK1* fusion protein was enrolled phase I clinical trial with larotrectinib and had nearly complete regression of the lung tumors. In the 2018 basket trial evaluating larotrectinib, 21/55 patients had sarcomas and all but 2 experienced at least a partial response [12]. In 2018, an integrated analysis of two phase I clinical trials and one phase II clinical trial revealed that entrectinib had a 57% ORR among 54 patients with NTRK fusions [15]. Entrectinib was well tolerated, produced durable systemic responses, and was FDA-approved based on these data.

In LPF-NT, the frontline therapy is surgical resection. However, in patients for whom surgery is not an option due to location or bulk of tumor, no chemotherapies or molecular therapies have successfully reduced tumor burden. To our knowledge, this case represents the first successful treatment of LPF-NT with an *NTRK*-fusion inhibitor prior to surgery. Our patient had an excellent response to entrectinib, allowing him to proceed to surgical resection. Importantly, post-operative pathology revealed > 95% necrosis, consistent with exquisite inhibitor sensitivity. These results suggest that all patients with a presumed diagnosis of LPF-NT should be screened for *NTRK* fusions using sensitive methodologies, and that treatment with a TRK fusion-protein inhibitor is a rational therapeutic option for patients who are not up-front surgical candidates.

## Data Availability

Not applicable.

## Abbreviations

LPF-NT: Lipofibromatosis-like neural tumor
NTRK: Neurotrophic receptor tyrosine kinase
MASC: Mammary analog of secretory carcinoma
NSCLC: Non-small cell lung cancer
NGS: Next generation sequencing
MPNST: Malignant peripheral nerve sheath tumor
